# Frequency of pathogenic germline variants in BRCA1, BRCA2, PALB2, CHEK2 and TP53 in ductal carcinoma in situ diagnosed in women under the age of 50 years

**DOI:** 10.1186/s13058-019-1143-y

**Published:** 2019-05-06

**Authors:** Christos Petridis, Iteeka Arora, Vandna Shah, Anargyros Megalios, Charlotte Moss, Anca Mera, Angela Clifford, Cheryl Gillett, Sarah E. Pinder, Ian Tomlinson, Rebecca Roylance, Michael A. Simpson, Elinor J. Sawyer

**Affiliations:** 1 School of Cancer and Pharmaceutical Sciences, Guy’s Hospital, King’s College London, London, SE1 9RT UK; 2 Medical and Molecular Genetics, Guy’s Hospital, King’s College London, London, SE1 9RT UK; 3 Institute of Cancer and Genomic Sciences, University of Birmingham, Edgbaston, Birmingham, B15 2TT UK; 4 Department of Oncology, UCLH Foundation Trust, London, NW1 2PG UK; 5 Innovation Hub, Guy’s Cancer Centre, Guy’s Hospital, London, SE1 9RT UK

**Keywords:** Germline variants, *BRCA2*, *CHEK2*, *PALB2*, *BRCA1*, *TP53*, Ductal carcinoma in situ

## Abstract

**Introduction:**

Ductal carcinoma in situ (DCIS) is a non-obligate precursor of invasive ductal breast cancer, and approximately 20% of screen-detected tumours are pure DCIS. Most risk factors for breast cancer have similar associations with DCIS and IDC; however, there is limited data on the prevalence of the known high and moderate penetrance breast cancer predisposition genes in DCIS and which women with DCIS should be referred for genetic screening.

The aim of this study was to assess the frequency of germline variants in *BRCA2*, *BRCA1*, *CHEK2*, *PALB2* and *TP53* in DCIS in women aged less than 50 years of age.

**Methods:**

After DNA extraction from the peripheral blood, Access Array technology (Fluidigm) was used to amplify all exons of these five known breast cancer predisposition genes using a custom made targeted sequencing panel in 655 cases of pure DCIS presenting in women under the age of 50 years together with 1611 controls.

**Results:**

Case-control analysis revealed an excess of pathogenic variants in *BRCA2* (OR = 27.96, 95%CI 6.56–119.26, *P* = 2.0 × 10^−10^) and *CHEK2* (OR = 8.04, 95%CI 2.93–22.05, *P* = 9.0 × 10^−6^), with weaker associations with *PALB2* (*P* = 0.003), *BRCA1* (*P* = 0.007) and *TP53* (*P* = 0.02). For oestrogen receptor (ER)-positive DCIS the frequency of pathogenic variants was 9% under the age of 50 (14% with a family history of breast cancer) and 29% under the age of 40 (42% with a family history of breast cancer). For ER-negative DCIS, the frequency was 9% (16% with a family history of breast cancer) and 8% (11% with a family history of breast cancer) under the ages of 50 and 40, respectively.

**Conclusions:**

This study has shown that breast tumourigenesis in women with pathogenic variants in *BRCA2*, *CHEK2*, *PALB2*, *BRCA1* and *TP53* can involve a DCIS precursor stage and that the focus of genetic testing in DCIS should be on women under the age of 40 with ER-positive DCIS.

**Electronic supplementary material:**

The online version of this article (10.1186/s13058-019-1143-y) contains supplementary material, which is available to authorized users.

## Introduction

Ductal carcinoma in situ (DCIS) is considered a non-obligate precursor of invasive breast cancer of ductal/no special type (IDC) as many IDCs have evidence of associated DCIS at presentation [1, 2] and the two components have similar genetic changes, suggesting that in the majority of cases the invasive component has arisen from the DCIS [3]. Synchronous DCIS is more frequent in luminal and HER2-positive IDC (53% and 63%, respectively) than invasive basal breast cancer (33%) [4]. Since the introduction of screening mammography, there has been an increase in the reported incidence of pure DCIS with no invasive component [5], with about 20% of screen-detected tumours being pure DCIS [6].

Most non-genetic risk factors for breast cancer have similar associations with DCIS and IDC, again supporting the notion that DCIS is a precursor of invasive cancer [7, 8]. Epidemiological studies have shown there is an inherited predisposition to DCIS, with women with DCIS being 2.4 times more likely to have an affected mother and sister with breast cancer than controls [9]. One study of almost 40,000 women suggested that the familial relative risk of DCIS may be greater than that of invasive breast cancer [10], but this was not confirmed in the Million Women Study, which showed a similar association with family history for DCIS and IDC [8].

The familial risk associated with invasive breast cancer is in part explained by both high-risk rare variants and low-risk susceptibility loci. We have shown that the majority of low-risk invasive breast cancer predisposition loci also predispose to DCIS and, as for invasive disease, different loci predispose to ER-positive and ER-negative DCIS and high and low grade DCIS [11]. However, the frequency of pathogenic high- and moderate-risk variants in DCIS is not clear. Claus et al. studied 369 women (mean age 53.8 years) with pure DCIS selected from a case-control study of carcinoma in situ and found that 2.4% had pathogenic variants in *BRCA2* and 0.8% in *BRCA1* [12]. Hall et al. analysed a highly selected cohort of women with carcinoma in situ (LCIS and DCIS) that were referred to Myriad for genetic testing. They found that 5.2% of women with pure carcinoma in situ (CIS) had *BRCA1/2* mutations (2.3% if women with a family history of breast cancer were excluded) and like Claus et al. that *BRCA2* mutations were more common than *BRCA1* [13]*.* Both these studies were performed before gene panel genetic testing was available, and therefore, other breast cancer predisposition genes such as *PALB2*, CHEK*2* and *TP53* were not assessed.

In this study, we report the frequency of rare variants in five known breast cancer predisposition genes (*BRCA2*, *BRCA1*, *TP53*, *CHEK2* and *PALB2)* in 655 cases of pure DCIS with no invasive disease in women diagnosed before the age of 50. These cases were included in our previous study of low-risk susceptibility loci in DCIS [11].

## Methods

### Samples

Six hundred fifty-five cases of pure DCIS with no invasive disease diagnosed in women aged under 50 were included in this study, Table 1. The majority of cases (633) were recruited through the ICICLE study (MREC 08/H0502/4) from 95 hospitals throughout the UK. This study was set up with the specific aim of investigating genetic predisposition to DCIS in the UK. A further 22 cases were recruited through the King’s Health Partners (KHP) Cancer Biobank (NHS REC ref. 12-EE-0493). Samples from patients under the age of 50 were selected for this analysis in order to enrich for cases likely to have a genetic component to their disease.

**Table 1:** ER status and cytonuclear grade of DCIS by age

Age	Total	ER+	ER−	ER unknown	High grade	Int grade	Low grade	Grade unknown
40–49	555	345 (62%)	62 (11%)	148 (27%)	345 (62%)	148 (27%)	47 (8%)	15 (3%)
< 40	100	45 (45%)	26 (26%)	29 (29%)	72 (72%)	20 (20%)	7 (7%)	1 (1%)
All	655	390	88	177	417	168	54	16

All controls were collected through the ICICLE and GLACIER studies (a similar study of lobular breast cancer, MREC 06/Q1702/64) and were identified by asking patients (cases) from both studies at the time of recruitment to identify female non-blood relatives or friends who were willing to donate a blood sample. These healthy volunteers were only eligible if they had no personal or family (up to second degree) history of invasive breast cancer, lobular carcinoma in situ (LCIS), DCIS or benign breast disease. Controls were not age matched and could be of any age, although older individuals were preferred (super-controls), as they had lived through many of their at-risk years.

All participants (cases and controls) donated a blood sample and were asked to complete a self-administered paper-based questionnaire on their family history.

Data on grade and oestrogen receptor (ER) status was ascertained mostly from the hospital pathology reports. In 200 cases where the grade data was missing from the report but a tumour block was available, a H&E section was cut and the DCIS was graded by the study histopathologist (SEP) according to UK and CAP guidelines [14]. A subset of 81 cases, graded in the pathology report and with a tumour block available, was examined to assess the reliability of the cytonuclear grade provided by the pathology reports. In the majority of cases (86.5%), grade was concordant with the pathology report. Nine cases were re-graded as low/intermediate grade and two cases as high grade. As the study pathologist re-graded the samples on a single H&E section, rather than all the blocks from an individual case, and in some cases on re-excision specimens with residual disease rather than the original excision specimen, the grade reported in the pathology report, if available, was used for the purposes of this study.

If ER status was not available from the local histopathology report and the tumour block was available, immunohistochemistry was performed and scored using the Allred method as previously described [11]. An Allred score of 3 or more was considered ER+ and that with scores of 0–2 (approximately equivalent to less than 1% of nuclei) were regarded as ER−.

### Next-generation sequencing

After DNA extraction from the peripheral blood, Access Array technology (Fluidigm) was used to amplify all exons of *BRCA2*, *BRCA1*, *TP53*, *CHEK2* and *PALB2* using a custom made targeted sequencing panel consisting of 321 amplicons (Additional file 1). The Fluidigm designed primers were supplied in single-plex with forward and reverse primers combined; these were multiplexed according to supplier’s instruction to achieve optimal efficiency. Purified libraries were quantified using Qubit High Sensitivity Assay Kit, and their average length size was measured in Tapestation using the D1000 screentape. The quantity and length size values obtained from the readings were used to calculate the final Molar concentration in order to prepare each sequencing library at 4 nM containing 960 samples, based on the following formula:

$$ \mathrm{Molar}\ \mathrm{Concentration}\ \left(\mathrm{in}\ \mathrm{nM}\right)=\mathrm{Concentration}\ \left(\mathrm{in}\ \mathrm{ng}\right)\times {10}^6\times \left(\frac{1}{649}\right)\times \left(\frac{1}{\mathrm{average}\ \mathrm{size}\ \left(\mathrm{in}\ \mathrm{bp}\right)}\right) $$

All quantified libraries were subsequently sequenced on a HiSeq2500 (Illumina).

### Bioinformatics analysis

Primer sites from the amplicons were trimmed using Btrim, and then, sequences were aligned to the reference genome (http://www.novocraft.com, GRCh37 version) using Novoalign (Gap opening penalty = 65 and gap extension penalty = 7 thresholds were applied). Picard tools (v1.74 https://github.com/broadinstitute/picard) and Bedtools (v2.17.0) were used to assess coverage. Variant calling was performed using Samtools and annotated using the Annovar tool [15]. We optimised the calling based on a set of variants that were positive controls, and Samtools was the optimal caller compared to HaplotypeCaller from GATK. The transcript that was used for each gene is reported in Additional file 1. The frequency of variant alleles from European reference populations was obtained from three sources (1000 genomes, ESP, ExAC), Additional file 2.

Variants were further filtered based on read depth, quality score, and genotypic quality. All variants with a read depth < 10, quality score < 20, or genotypic score < 20 were excluded from the analysis. In addition, variants with an allelic ratio < 0.2 were excluded irrespective of read depth and variants with an allelic ratio < 0.3 and read depth < 50 were also removed.

Variants that had been previously clinically evaluated and deposited in the ClinVar database (https://www.ncbi.nlm.nih.gov/clinvar/) were assigned labels of benign, variants of unknown significance (VUS), conflicting or pathogenic, as per ClinVar.

Variants not present in the ClinVar database were considered pathogenic if they were predicted to lead to protein truncation (frameshift indels, stop-gain, stop-loss or intronic variants within two base pairs of the splicing junction; Additional file 3) and variants of unknown significance (VUS) if they were nonsynonymous substitutions or in-frame indels. Novel variants in the last exon of a gene meeting the above criteria of pathogenic were not excluded as, although unlikely to result in loss of function through nonsense mediated decay, these variants may have substantial impact on the protein product. However, no such variants were detected in this study.

To further investigate the importance and validity of our findings, we used an external resource of controls. A non-Finnish European population of controls from gnomAD (gnomAD controls v2.1) was used as a replication control cohort.

### Statistical analysis

Fisher’s exact test was used for gene based rare variant analysis for both case-control and case-only analyses. One sided test was selected since the expectation was enrichment rather than deficit of variants in cases over controls. No adjustments have been made to account for multiple testing. With the current sample size, we have ~ 80% power (alpha =0.05) to detect variants of combined allele frequency = 0.001 and an effect size of OR = 5.

### Validation

All putative pathogenic alleles identified by the above methods were confirmed by Sanger sequencing.

### Assessment of copy number variation

Two bioinformatics tools were used to assess copy number variation (CNV) in our sequencing data: CNVkit version 0.9.5 [16] and ONCOCNV version 6.9 [17]. The first, CNVkit, running in amplicon sequencing mode, was used to identify exon-level copy number variations using on-target coverages alone. One thousand six hundred eleven control samples were used to construct a reference copy number profile, and the default circular binary segmentation (CBS) algorithm was used to derive segments. The second, ONCOCNV, is a package specifically designed for amplicon sequencing data. Fifteen randomly selected control samples were used to construct the reference copy number profile, and the default cghseg algorithm was used to derive segments. All CNVs detected by these methods underwent multiplex ligation-dependent probe amplification (MLPA) using MRC Holland kits (https://www.mlpa.com/) in order to validate the finding. DNA samples were amplified, and PCR products were analysed on the ABI 3730 Genetic Analyser (Applied Biosystems) using TAMRA 500, as per the manufacturer’s instructions. Coffalyser (MRC Holland) was used to call exonic deletions/rearrangements.

## Results

The analysis was performed on 655 cases of pure DCIS with no invasive disease diagnosed in women aged under 50, together with 1611 controls. The median age of cases was 45 years (interquartile range 6) and of controls was 52 (interquartile range 12). Data on grade and oestrogen receptor (ER) status were available for 98% and 73% of the cases in the study, respectively, Table 1.

The mean coverage of our target region was 800 reads across all samples, with an average of at least 40 reads for more than 98% of the target region per sample. Of the 321 amplicons analysed, seven failed to amplify consistently across the majority of the samples; however, even for these seven the majority of samples had at least 10 reads for 90% of the amplicon, and there was no difference in amplification between cases and controls, Additional file 4. No exon-level CNVs were identified using CNVkit version 0.9.5. Thirteen copy number variants across eight samples were detected by ONCOCNV; however, none were confirmed using MLPA, Additional file 5.

We found an association with DCIS and pathogenic variants in *BRCA2*, *CHEK2*, *PALB2*, *BRCA1* and *TP53*, Table 2 (individual raw data, Additional file 2).

**Table 2:** Association of known pathogenic variants and DCIS in women < 50 years of age by gene. For the GnomAD comparison, Non-Finnish European controls were used (v2.1)

Gene	Pathogenic variants in cases (*N* = 655)	Pathogenic variants in controls (*N* = 1611)	OR (95% CI)	*P* value	Pathogenic variants in gnomAD controls (*N* = 21,384)	OR (95% CI) (vs gnomAD controls)	*P* value (vs gnomAD controls)
*BRCA2*	22 (3.4%)	2 (0.1%)	27.96 (6.56–119.26)	2 × 10^−10^	76 (0.35%)	9.74 (6.02–15.76)	1 × 10^−13^
*CHEK2*	16 (2.4%)	5 (0.3%)	8.04 (2.93–22.05)	9 × 10^−6^	144 (0.67%)	3.69 (2.19–6.23)	2 × 10^−5^
*PALB2*	6 (0.9%)	1 (0.06%)	14.88 (1.79–123.88)	0.003	22 (0.1%)	8.98 (3.63–22.21)	1 × 10^−4^
*BRCA1*	4 (0.6%)	0 (0%)	inf	0.007	81 (0.38%)	1.62 (0.59–4.42)	0.32
*TP53*	3 (0.5%)	0 (0%)	inf	0.02	16 (0.07%)	6.14 (1.79–21.14)	0.018

### BRCA2

**Fig. 1:**
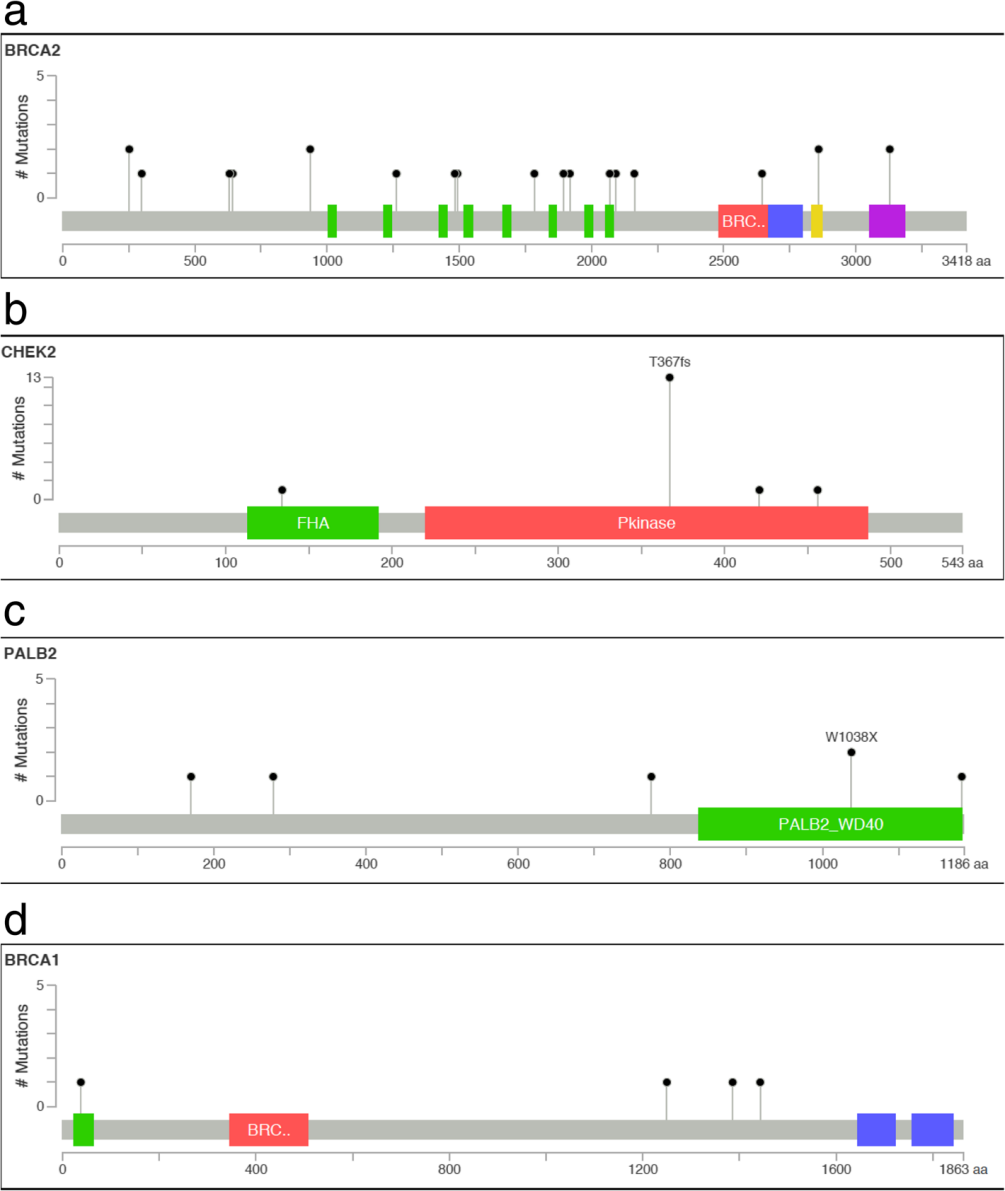
Position of pathogenic variants in DCIS cases in **a** *BRCA2*, **b** *CHEK2*, **c** *PALB2*, **d** *BRCA1*

The strongest association was with *BRCA2* (OR = 27.96, 95%CI 6.56–119.26, *P* = 2 × 10^−10^), Table 2, Fig. 1a. Of the 22 pathogenic variants identified, all had been previously described apart from a novel frameshift in exon 11 (c.5754dupT:p.H1918fs). Fifty percent of pathogenic variants occurred in exon 11. Only two pathogenic variants occurred in more than one patient: exon20:c.8575delC:p.Q2859fs in two patients and exon25:c.C9382T:p.R3128X in two patients, Additional file 6. Ninety-five percent of the cases with a pathogenic variant in *BRCA2* had high or intermediate grade DCIS, and in the 15 cases where ER status was known, all were ER positive. There was also an association with age < 40 years (OR = 4.12, 95%CI 1.75–9.69, *P* = 0.003), Table 3, and family history of breast cancer in a first-degree relative (OR = 4.29, 95%CI 1.82–10.08, *P* = 0.001), Table 4.

**Table 3:** Case only analysis of pathogenic variants in DCIS by age

Gene	Pathogenic variants in cases aged < 40 (*N* = 100)	Pathogenic variants in cases aged 40–49 (*N* = 555)	OR (95% CI)	*P* value
*BRCA2*	9 (9%)	13 (2.3%)	4.12 (1.75–9.69)	0.003
*CHEK2*	3 (3%)	13 (2.3%)	1.29 (0.37–4.54)	0.72
*PALB2*	1 (1%)	5 (0.9%)	1.11 (0.13–9.53)	1
*BRCA1*	1 (1%)	3 (0.5%)	1.86 (0.19–17.90)	0.485
*TP53*	1 (1%)	2 (0.4%)	2.79 (0.25–30.86)	0.39

**Table 4:** Case only analysis of pathogenic variants in DCIS for family history of breast cancer

Gene	Carriers with FH of BC in first degree relative vs no FH		Carriers with FH of BC in any relative vs no FH	
	OR (95% CI)	*P*	OR (95% CI)	*P*
*BRCA2*	4.29 (1.82–10.08)	0.001	4.90 (1.65–14.54)	0.002
*CHEK2*	0.93 (0.30–2.90)	1	1.76 (0.64–4.86)	0.32
*PALB2*	14.37 (1.68–123.25)	0.006	5.27 (0.61–45.20)	0.12
*BRCA1*	8.52 (0.88–82.07)	0.058	3.14 (0.33–30.26)	0.36
*TP53*	1.40 (0.13–15.47)	1	2.09 (0.19–23.07)	0.62

*FH* family history, *BC* breast cancer

### CHEK2

*CHEK2* also showed a strong association with DCIS (OR = 8.04, 95%CI 2.93–22.05, *P* = 9 × 10^− 6^), but with a smaller effect size than *BRCA2*, Table 2, Fig. 1b. Of the 16 pathogenic variants detected, 13 were the c.1100delC mutation. The remaining three variants were novel frameshift mutations, one in exon 3: c.401_402del, and two in exon 12: c.1262delT and c.1368dupA, Additional file 7. Again, all were high or intermediate grade DCIS and, of the 14 with known ER status, 12 were ER positive. There was no association with age < 40 (81% of cases occurred in the 40–49 age group) or family history, Tables 3 and 4.

### PALB2

There was an association with *PALB2* (OR 14.88, 95%CI 1.79–123.88, *P* = 0.003), Table 2, but this was weaker than that for *BRCA*2 and *CHEK2*. Of the six pathogenic *PALB2* variants detected, three were novel frameshifts (two in exon 4, one in exon 5, Fig. 1c, Additional file 8). Of the three known, two were in exon 10 (rs180177132), which has previously been shown to be associated with breast cancer (OR = 4.21, 95%CI 1.85–9.61), but with no evidence of a differential association with ER status [18]. All women with *PALB2* pathogenic variants had high or intermediate grade DCIS and were ER positive. There was no association with age < 40, but there was an association with family history of breast cancer in a first-degree relative (OR = 14.37, 95%CI 1.68–123.25, *P* = 0.006), Table 4.

### BRCA1

There were four DCIS cases that were found to harbour pathogenic *BRCA1* germline variants and no controls ((*P* = 0.007), Table 2, Additional file 9. One was the well-described exon12:c.C4327T:p.R1443X, and the other three were novel frameshift mutations, two in exon 11 and one in exon 3. Three were ER negative (one unknown) and three high-grade DCIS. There was no association with age < 40 and a borderline association with family history of breast cancer in a first-degree relative (*P* = 0.058). Three of the cases had a family history of breast and or ovarian cancer in first-degree relatives under the age of 50. However, the case with the novel frameshift variant in exon 10: c.3750delG:p.E1250fs had a strong family history of other cancers (cervical, lung, and oral) but not breast.

### TP53

There were three DCIS cases that were found to harbour pathogenic *TP53* germline variants and no controls ((*P* = 0.02), Table 2. Two were stopgain variants (one novel), and the other a nonsynomous variant (rs397514495) considered pathogenic in the literature [19], but recently suggested to be a VUS as it does not disrupt all the functions of TP53 just apoptosis [20], Additional file 10a. The women that carried these variants did not meet the criteria for classic Li-Fraumeni or Li-Fraumeni-like syndrome. The novel stopgain variant (NM_000546:exon4:c.G272A:p.W91X) was found in a woman with bilateral DCIS at age 35 and a first-degree relative with breast cancer < 40 years, but no other cancers. The other known stopgain variant was identified in a woman with DCIS at age 40 whose father developed an unknown cancer at age 50 but with no other family history of cancer. The missense variant occurred in a woman with DCIS at age 45 and a family history of breast cancer in a second-degree relative aged 60, but no other cancers. Again, all had intermediate or high-grade DCIS, with two ER positive and two ER negative (in the bilateral case one side was ER positive and the other ER negative).

As most pathogenic germline mutations in *TP53* are missense rather than truncating mutations [21], it is possible that by only considering novel variants as pathogenic if they were predicted to lead to protein truncation we may have missed novel pathogenic missense variants in *TP53*. We therefore looked at these in more detail. Only one novel missense variant was detected, NM_000546:exon10:c.G1054 T:p.D352Y, and this was in a control. Three variants called as VUS in the ClinVar database were identified in cases, Additional file 10b. One NM_000546:exon8:c.G869A:p.R290H is listed in the IARC *TP53* database [22] as being associated with Li-Fraumeni–like syndrome and, in our study, was found in one case and two controls.

### Biallelic mutation carriers

There were four patients carrying two pathogenic variants in different genes. All had a family history of cancer and three a family history of breast cancer, Additional file 11.

### Bilateral and subsequent invasive disease

Metachronous bilateral disease was most commonly found in *CHEK2* mutation carriers with 5/16 (31%) developing subsequent contralateral disease after a diagnosis of unilateral DCIS (in two, this was in the form of an invasive disease, in two LCIS and in one DCIS) compared to 2/22 (9%) for *BRCA2.* The latter, however, will be influenced by the fact that nine of the *BRCA2* carriers elected for bilateral prophylactic mastectomies based on their family history or the results of genetic testing (five underwent genetic testing). As this study does not have long-term follow-up data, it was not possible to assess whether germline pathogenic variants were associated with subsequent invasive recurrence of DCIS.

### Variants of unknown significance

Analysis of variants of unknown significance (VUS), either known or novel, revealed no excess of VUS in *BRCA2*, *PALB2*, *TP53* or *BRCA1*, Additional file 12. A VUS in exon 4 of *CHEK2* (rs77130927, c.C538T:p.R180C), which we have previously shown to have a borderline association with invasive lobular cancer (ILC) (*P* = 0.03, Petridis et al, accepted *Cancer Epidemiology, Biomarkers & Prevention*), was found in two DCIS cases and one control.

## Discussion

Early data on women with *BRCA1* and *BRCA2* mutations suggested that presentation as pure DCIS was infrequent. However, Yang et al. showed that ~ 60% of *BRCA1*- and *BRCA2*-associated invasive tumours had associated DCIS of similar phenotype [23]. They also found that the number of pure DCIS cases was similar in *BRCA1* and *BRCA2* mutations carriers (21% vs 23%), in contrast to Krammer et al. who reported that pure DCIS was more frequent in *BRCA2* mutation carriers compared to *BRCA1* carriers (5%, 36/246, versus 9%, 23/250, *P* = 0.0026) [24]. In our study of sporadic pure DCIS, pathogenic *BRCA2* variants were far more common than *BRCA1* mutations (3.5% vs 0.6%). This is similar to the data of Claus et al. [12] who found that 2.4% had pathogenic variants in *BRCA2* and 0.8% in *BRCA1* in a slightly older group of DCIS patients (mean age 53.8 years). Similarly, Hall et al. found that 5.2% of women under 50 years of age with carcinoma in situ (LCIS and DCIS) had *BRCA1/2* mutations, with *BRCA2* mutations being more common than *BRCA1* [13].

We found that *BRCA2* mutations occurred in 2.4% of DCIS in women under the age 50 and in 9% under the age of 40. All but one of these variants had been previously described in invasive breast cancer. Of the genes studied, *BRCA2* was the only gene where pathogenic mutations were associated with younger age. All the cases of DCIS in *BRCA2* carriers were ER positive (where ER status was known), unlike invasive disease where only 77% are ER positive [25]. In contrast, *BRCA1* pathogenic variants were infrequent, four in total (only one had been previously described), and associated with predominantly ER negative DCIS.

Pathogenic variants in *CHEK2* were the second most common set of mutations after *BRCA2* and occurred in 2.5% of pure DCIS under the age of 50. Unlike *BRCA2*, there was no association with age and the majority were the well-described c.1100delC variant. There was no evidence of an association with the rare missense variant p.I157T (c.T470C, rs17879961), which was found in three controls and no cases. This high frequency of *CHEK2* variants in pure DCIS has not been previously described, although Schmidt et al. noted in their study of tumour characteristics in *CHEK2* c.1100delC carriers that carriers from population- and hospital-based studies more often developed in situ tumours (LCIS and DCIS) compared to carriers from familial or clinical genetics center–based studies; this was interpreted as a bias estimate due to differential recruitment related to family history of breast cancer and screening [26]. Our findings are also supported by Couch et al. who reported data on the frequency of *CHEK2* mutations in a series of breast cancer with and without pure DCIS allowing one to determine mutation rates in pure DCIS cases. In that study, 2.87% of DCIS cases had pathogenic *CHEK2* variants compared to 1.43% in invasive disease [27].

The tumour phenotypes associated with *PALB2* tumours are very similar to those associated with *BRCA2* tumours, with 61% of invasive tumours having associated DCIS [28]. It is therefore not surprising that we have found *PALB2* pathogenic mutations in women with pure DCIS and that they are more common in women with a first-degree relative with breast cancer. However, unlike *BRCA2*, they were not associated with age < 40 years, this is supported by the findings of Antoniou et al. who showed there was a constant relative risk, irrespective of age, for pathogenic variants in *PALB2* [29]. The frequency of *PALB2* variants in our data is supported by the study by Couch et al. where one can infer from the supplementary data that the frequency of pathogenic *PALB2* variants in DCIS is 0.96% [27].

Pure DCIS (73% HER2 positive, 55% ER positive) and high-grade comedo DCIS have been described in Li-Fraumeni Syndrome [30, 31] but our data show that *TP53* mutations are infrequent in sporadic DCIS. Although two of three pathogenic variants identified had been previously described, none of the women had a family history of cancer to suggest Li-Fraumeni Syndrome (LFS).This may be because they are de novo mutations or because two were loss of function mutations which often do not have such a typical LFS phenotype as dominant negative missense mutations [32]. The known missense mutation detected has been shown to be associated with Li-Fraumeni–like syndrome rather than true LFS [22].

The odds ratios presented in this study are higher than those previously reported for these genes in invasive disease. This is likely due to the size of the study which is too small to yield stable estimates of associations with DCIS, but does give useful estimates of prevalence and, of note, is twice as large as the study by Claus et al., the only other study documenting the prevalence of *BRCA1/2* mutations in sporadic DCIS. The other reason is due to the use of older controls. When we compare our cases to 21,384 non-Finnish European controls from gnomAD (gnomAD controls v2.1, http://gnomad.broadinstitute.org), we see that the odds ratios fall to similar levels previously reported for invasive cancer with the exception of *CHEK2* (OR = 3.69, 95%CI.19–6.23) which still remains higher than that reported in studies of invasive breast cancer (OR~ 2), Table 2. Schmidt et al. also found this in their large series from the Breast Cancer Association Consortium (invasive: OR = 2.4, 95%CI 2.04–2.82, in situ: OR = 3.53, 95%CI 2.38–5.23) [26]). We also found a similar finding in lobular cancer where LCIS had a stronger association with *CHEK2* mutations than ILC (ILC OR = 4.29, 95%CI 1.60–11.51, *P* = 0.0017; LCIS OR = 9.95, 95%CI 3.44–28.82, *P* = 5 × 10^−5^, Petridis et al. accepted *Cancer Epidemiology, Biomarkers & Prevention*). This suggests that *CHEK2* pathogenic variants may be predisposing to the in situ stage of breast cancer with some not progressing to the invasive state.

In a study of 6478 patients with invasive breast cancer under the age of 50, Schmidt et al. [33] found a higher frequency of *BRCA1* mutations compared to our study of DCIS (3.2% versus 0.6%). We believe that this difference in frequency of *BRCA1* mutations stems from the fact that the vast majority of the samples in our study are ER+ and only 13% of the samples are ER−, compared to 25% in the invasive study of Schmidt et al.

There is currently a debate as to the need for mutation screening in women with DCIS. In this study, 7.2% of women with DCIS (irrespective of ER status) under the age of 50 had pathogenic variants in one of five known breast cancer predisposition genes. This level does not reach the current UK threshold for genetic testing (https://www.nice.org.uk/guidance/cg164/chapter/Recommendations#genetic-testing); however, women under 40 years of age had a 13% (11% excluding *CHEK2* variants) probability of having a germline mutation which does reach the UK threshold of 10% for routine testing. For women under 40 years of age with a family history of breast cancer, the frequency of germline mutations increases to 21%.

There has been particular focus on ER-negative DCIS and whether these women should undergo HER2 testing and, if this is also negative, be offered *BRCA1* and *BRCA2* testing, as is recommended for those with triple-negative invasive breast cancer. Unfortunately, in our series, we did not have data on HER2 receptor status as it is not routinely assessed in cases of DCIS in present clinical practice. However, looking solely at ER-negative DCIS, only 9% under the age of 50 and 8% under the age of 40 had pathogenic variants and these were in *BRCA1*, *TP53 and CHEK2*. These figures rise to 16% and 11%, respectively, if only women with a family history of breast cancer are considered, Table 5. In contrast, the frequency of pathogenic variants in ER-positive DCIS under the age of 40 was much higher; 9% of women under the age of 50 had pathogenic variants rising to 29% under the age of 40 (14% and 42%, respectively, if only women with a family history of breast cancer are considered) Table 6.

**Table 5:** Frequency of pathogenic variants in ER-negative DCIS by age

Gene	Frequency under 40 years *N* = 26	Frequency under 40 years with FH *N* = 9	Frequency under 50 years *N* = 89	Frequency under 50 years with FH *N* = 38
*BRCA2*	0 (0%)	0 (0%)	0 (0%)	0 (0%)
*CHEK2*	1 (3.8%)	0 (0%)	3 (3.3%)	2 (5.2%)
*PALB2*	0 (0%)	0 (0%)	0 (0%)	0 (0%)
*BRCA1*	0 (0%)	0 (0%)	3 (3.3%)	3 (7.9%)
*TP53*	1 (3.8%)	1 (11%)	2 (2.2%)	1 (2.6%)
Total (% of ER− cases)	2 (8%)	1 (11%)	8 (9%)	6 (16%)

*FH* family history of breast cancer in any relative

**Table 6:** Frequency of pathogenic variants in ER-positive DCIS by age

Gene	Frequency under 40 years *N* = 45	Frequency under 40 years with FH *N* = 24	Frequency under 50 years *N* = 397	Frequency under 50 years with FH *N* = 197
*BRCA2*	8 (17.8%)	7 (29.2%)	16 (4%)	14 (7.1%)
*CHEK2*	2 (4.4%)	1 (4.2%)	12 (3%)	7 (3.6%)
*PALB2*	1 (2.2%)	1 (4.2%)	5 (1.3%)	4 (2%)
*BRCA1*	1 (2.2%)	0 (0%)	1 (0.3%)	0 (0%)
*TP53*	1 (2.2%)	1 (4.2%)	2 (0.5%)	2 (1%)
Total (% of ER+ cases)	13 variants (29%) in 11 women (24%)*	10 variants (42%) in 9 women (38%)	36 variants (9%) in 32 women (8%)	27 variants (14%) in 24 women (12%)

*FH* family history of breast cancer in any relative

*Some individuals carry 2 variants

## Conclusions

This study has shown that a DCIS-associated malignant pathway can occur in patients who have pathogenic variants in *BRCA2*, *CHEK2*, *PALB2*, *BRCA1* and *TP53*. We also show that the focus of genetic testing should be on ER-positive, intermediate-, and high-grade DCIS from patients under the age of 40, rather than ER-negative DCIS, although restricting such testing to those age under 40 would fail to identify the majority of *CHEK2* and *PALB2* mutation carriers. Once mutations are identified in these women, chemoprevention with tamoxifen and surveillance is a potential alternative to prophylactic mastectomy, particularly in *CHEK2* carriers where the risk of invasive disease is less. Further studies with long-term follow-up data are required to ascertain whether these germline pathogenic variants identify a subgroup of DCIS that are more likely to progress to invasive disease or whether somatic changes in the DCIS are a more important predictor of recurrence.

## Additional files

Additional file 1: Targeted sequencing panel. (DOCX 19 kb)

Additional file 2: Coverage data for all pathogenic variants identified. (XLSX 14 kb)

Additional file 3: Definition of variants. (DOCX 18 kb)

Additional file 4: Amplicons that failed to amplify consistently. (DOCX 19 kb)

Additional file 5: Copy number variation detected by ONCOCNV. (DOCX 15 kb)

Additional file 6: *BRCA2* pathogenic variants in cases. (DOCX 21 kb)

Additional file 7: *CHEK2* pathogenic variants in cases. (DOCX 20 kb)

Additional file 8: *PALB2* pathogenic variants in cases. (DOCX 19 kb)

Additional file 9: *BRCA1* pathogenic variants in cases. (DOCX 18 kb)

Additional file 10: a: *TP53* pathogenic variants in cases. b: *TP53* VUS in cases. (DOCX 20 kb)

Additional file 11: Age of onset of DCIS and family history details of women with two pathogenic variants in different genes. (DOCX 19 kb)

Additional file 12: Frequency of variants of unknown significance and DCIS in women < 50 years of age by gene. (DOCX 19 kb)

## Abbreviations

95 CI: 95% confidence interval
DCIS: Ductal carcinoma in situ
IDC: Invasive ductal cancer.
ILC: Invasive lobular cancer
LCIS: Lobular carcinoma in situ
OR: Odds ratio
VUS: Variant of unknown significance
