# Supplementary material for: Frequency of pathogenic germline variants in BRCA1, BRCA2, PALB2, CHEK2 and TP53 in ductal carcinoma in situ diagnosed in women under the age of 50 years
Source: Breast Cancer Res. 2019 May 6;21:58. doi: 10.1186/s13058-019-1143-y (PMC6501320; doi:10.1186/s13058-019-1143-y)
Supplement: Supplementary file 1 — Targeted sequencing panel. (DOCX 19 kb) [file 13058_2019_1143_MOESM1_ESM.docx]

Additional File 1: Targeted Sequencing Panel

| **Gene** | **Transcript** | **Length in bp** | **Total amplicons in gene** | **Exons** | **Amplicons** | **Coverage** |
| --- | --- | --- | --- | --- | --- | --- |
| *BRCA2* | NM_000059 | 10,257 | 126 | 28 | 126 | Full |
| *BRCA1* | NM_007300 | 5,552 | 68 | 24 | 68 | Full |
| *CHEK2* | NM_007194 | 1,761 | 28 | 22 | 28 | Full |
| *PALB2* | NM_024675 | 3,561 | 48 | 15 | 48 | Full |
| *TP53* | NM_000546.4 | 1,146 | 15 | 10 | 15 | Full |
