# Supplementary material for: Frequency of pathogenic germline variants in BRCA1, BRCA2, PALB2, CHEK2 and TP53 in ductal carcinoma in situ diagnosed in women under the age of 50 years
Source: Breast Cancer Res. 2019 May 6;21:58. doi: 10.1186/s13058-019-1143-y (PMC6501320; doi:10.1186/s13058-019-1143-y)
Supplement: Supplementary file 3 — Definition of variants. (DOCX 18 kb) [file 13058_2019_1143_MOESM3_ESM.docx]

Additional File 3: Definition of variants

.

| **Variant class** | **Variant type** | **Description** |
| --- | --- | --- |
| Missense/ non-synonymous | Protein altering | A single nucleotide substitution that leads to an amino-acid substitution |
| Stop-gain | Protein truncating | A single nucleotide substitution that leads to the introduction of a premature stop codon |
| Stop-loss | Protein truncating | A single nucleotide substitution that leads to the loss of the wild type stop codon |
| Frameshift indel | Protein truncating | An insertion or deletion of a number of nucleotides that leads to a frame-shift of the amino-acid sequence |
| Non-frameshift indel | Protein altering | An insertion or deletion of a number of nucleotides that leads to the addition or deletion of a number of amino-acids |
| Splicing | Protein truncating | A single nucleotide substitution in the essential splice site 1 or 2 nucleotides adjacent to the splice site |
| Synonymous | Silent | A single nucleotide substitution that leads to the same amino-acid being encoded |
