# Supplementary material for: Frequency of pathogenic germline variants in BRCA1, BRCA2, PALB2, CHEK2 and TP53 in ductal carcinoma in situ diagnosed in women under the age of 50 years
Source: Breast Cancer Res. 2019 May 6;21:58. doi: 10.1186/s13058-019-1143-y (PMC6501320; doi:10.1186/s13058-019-1143-y)
Supplement: Supplementary file 4 — Amplicons that failed to amplify consistently. (DOCX 19 kb) [file 13058_2019_1143_MOESM4_ESM.docx]

Additional File 4: Amplicons that failed to consistently amplify

| **Amplicon ID** | **Exonic region** | **% of samples with <10 reads for 90% of the exon** | **Amplicon size** | **% GC** | **Comment** |
| --- | --- | --- | --- | --- | --- |
| BRCA1_t6_5 | chr17:41251782-41251907 | 8.6% | 195 | 44 | Designed without SNP and repeat annotation |
| CHEK2_t1_1 | chr22:29130381-29130719 | 22.8% | 191 | 47 | Primers designed within a repeat region |
| CHEK2_t1_3 | chr22:29121221-29121365 | 0.1% | 185 | 58 |  |
| PALB2_t13_3 | chr16:23614770-23615000 | 0.1% | 200 | 41 |  |
| PALB2_t4_21 | chr16:23646173-23647665 | 0.2% | 180 | 21 |  |
