# Supplementary material for: Frequency of pathogenic germline variants in BRCA1, BRCA2, PALB2, CHEK2 and TP53 in ductal carcinoma in situ diagnosed in women under the age of 50 years
Source: Breast Cancer Res. 2019 May 6;21:58. doi: 10.1186/s13058-019-1143-y (PMC6501320; doi:10.1186/s13058-019-1143-y)
Supplement: Supplementary file 5 — Copy number variation detected by ONCOCNV. (DOCX 15 kb) [file 13058_2019_1143_MOESM5_ESM.docx]

Additional File 5: Exon level deletions detected by ONCOCNV

| **Gene** | **Variant** | **Number of samples with CNV** | **Previously Described** | **Validated by MLPA** |
| --- | --- | --- | --- | --- |
| *BRCA1* | Exon 2 deletion | 2 | Previously described as pathogenic | No |
| *BRCA2* | Exon 2 deletion | 1 | Previously described as pathogenic | No |
| *BRCA2* | Exon 12 deletion | 2 | Known to be functionally redundant | No |
| *CHEK2* | Exon 14 deletion | 7 | Previously described in Polish population in lymphoproliferative disease | No |
| *TP53* | Exon 11 deletion | 1 | No | No |
