# Supplementary material for: Frequency of pathogenic germline variants in BRCA1, BRCA2, PALB2, CHEK2 and TP53 in ductal carcinoma in situ diagnosed in women under the age of 50 years
Source: Breast Cancer Res. 2019 May 6;21:58. doi: 10.1186/s13058-019-1143-y (PMC6501320; doi:10.1186/s13058-019-1143-y)
Supplement: Supplementary file 6 — BRCA2 pathogenic variants in cases. (DOCX 21 kb) [file 13058_2019_1143_MOESM6_ESM.docx]

Additional File 6: *BRCA2* pathogenic variants in cases

| **Type of Mutation** | **Details** | **ID** | **Age** | | **Grade** | **ER status** | **Bilateral** |
| --- | --- | --- | --- | --- | --- | --- | --- |
| splicing | BRCA2:NM_000059:exon7:c.631+2T>G | RS81002899 | | 46 | Low | Positive | Yes |
| frameshift deletion | BRCA2:NM_000059:exon9:c.755_758del:p.D252fs, | rs80359659 | | 48 | High | Positive |  |
| frameshift deletion | BRCA2:NM_000059:exon9:c.755_758del:p.D252fs, | rs80359659 | | 39 | High | Missing |  |
| frameshift deletion | BRCA2:NM_000059:exon10:c.896_899del:p.V299fs, | Known_indel | | 38 | High | Positive |  |
| frameshift deletion | BRCA2:NM_000059:exon11:c.1929delG:p.V643fs, | RS80359316 | | 41 | High | Missing |  |
| frameshift deletion | BRCA2:NM_000059:exon11:c.2808_2811del:p.K936fs, | rs80359352 | | 43 | High | MISSING | Yes |
| frameshift deletion | BRCA2:NM_000059:exon11:c.2808_2811del:p.K936fs, | rs80359352 | | 49 | Intermediate | Positive |  |
| stopgain | BRCA2:NM_000059:exon11:c.C3785G:p.S1262X, | RS80358620 | | 37 | Intermediate | Positive |  |
| frameshift deletion | BRCA2:NM_000059:exon11:c.4447delA:p.T1483fs, | rs80359448 | | 38 | High | Positive |  |
| frameshift deletion | BRCA2:NM_000059:exon11:c.4478_4481del:p.E1493fs, | rs80359455 | | 44 | Intermediate | Positive |  |
| frameshift deletion | BRCA2:NM_000059:exon11:c.5350_5351del:p.N1784fs, | rs80359507 | | 41 | High | MISSING |  |
| stopgain | BRCA2:NM_000059:exon11:c.C5682G:p.Y1894X, | RS41293497 | | 30 | High | Positive |  |
| frameshift insertion | BRCA2:NM_000059:exon11:c.5754dupT:p.H1918fs, | NOVEL | | 47 | Intermediate | MISSING |  |
| stopgain | BRCA2:NM_000059:exon11:c.T6206G:p.L2069X, | RS80358859 | | 47 | Intermediate | MISSING |  |
| frameshift deletion | BRCA2:NM_000059:exon11:c.6275_6276del:p.L2092fs, | rs11571658 | | 38 | MISSING | MISSING |  |
| frameshift deletion | BRCA2:NM_000059:exon11:c.6486_6489del:p.K2162fs, | rs80359598 | | 47 | High | Positive |  |
| frameshift deletion | BRCA2:NM_000059:exon17:c.7934delG:p.R2645fs, | RS80359688 | | 32 | High | Positive |  |
| splicing | BRCA2:NM_000059:exon18:c.7977-1G>C | RS81002874 | | 34 | Intermediate | Positive |  |
| frameshift deletion | BRCA2:NM_000059:exon20:c.8575delC:p.Q2859fs, | RS80359718 | | 44 | Intermediate | Positive |  |
| frameshift deletion | BRCA2:NM_000059:exon20:c.8575delC:p.Q2859fs, | RS80359718 | | 40 | High | Positive |  |
| stopgain | BRCA2:NM_000059:exon25:c.C9382T:p.R3128X, | RS80359212 | | 42 | High | Positive |  |
| stopgain | BRCA2:NM_000059:exon25:c.C9382T:p.R3128X, | RS80359212 | | 34 | High | Positive |  |
