# Supplementary material for: Frequency of pathogenic germline variants in BRCA1, BRCA2, PALB2, CHEK2 and TP53 in ductal carcinoma in situ diagnosed in women under the age of 50 years
Source: Breast Cancer Res. 2019 May 6;21:58. doi: 10.1186/s13058-019-1143-y (PMC6501320; doi:10.1186/s13058-019-1143-y)
Supplement: Supplementary file 7 — CHEK2 pathogenic variants in cases. (DOCX 20 kb) [file 13058_2019_1143_MOESM7_ESM.docx]

Additional File 7: *CHEK2* pathogenic variants in cases

| **Type of Mutation** | **Details** | **ID** | **Age** | **Grade** | **ER status** | **Bilateral** |
| --- | --- | --- | --- | --- | --- | --- |
| frameshift deletion | CHEK2:NM_007194:exon11:c.1100delC:p.T367fs | RS555607708 | 38 | MISSING | MISSING |  |
| frameshift deletion | CHEK2:NM_007194:exon11:c.1100delC:p.T367fs | RS555607708 | 49 | High | Positive |  |
| frameshift deletion | CHEK2:NM_007194:exon11:c.1100delC:p.T367fs | RS555607708 | 43 | High | MISSING |  |
| frameshift deletion | CHEK2:NM_007194:exon11:c.1100delC:p.T367fs | RS555607708 | 44 | Intermediate | Positive |  |
| frameshift deletion | CHEK2:NM_007194:exon11:c.1100delC:p.T367fs | RS555607708 | 49 | Intermediate | Positive |  |
| frameshift deletion | CHEK2:NM_007194:exon11:c.1100delC:p.T367fs | RS555607708 | 44 | High | Negative |  |
| frameshift deletion | CHEK2:NM_007194:exon11:c.1100delC:p.T367fs | RS555607708 | 45 | Intermediate | Positive |  |
| frameshift deletion | CHEK2:NM_007194:exon11:c.1100delC:p.T367fs | RS555607708 | 46 | High | Positive |  |
| frameshift deletion | CHEK2:NM_007194:exon11:c.1100delC:p.T367fs | RS555607708 | 37 | High | Positive | Yes: contralateral DCIS + invasive, Subsequent unilateral Invasive |
| frameshift deletion | CHEK2:NM_007194:exon11:c.1100delC:p.T367fs | RS555607708 | 49 | High | Positive | Yes: contralateral LCIS |
| frameshift deletion | CHEK2:NM_007194:exon11:c.1100delC:p.T367fs | RS555607708 | 30 | High | Positive |  |
| frameshift deletion | CHEK2:NM_007194:exon11:c.1100delC:p.T367fs | RS555607708 | 48 | High | Positive | Yes: contralateral DCIS + invasive |
| frameshift deletion | CHEK2:NM_007194:exon11:c.1100delC:p.T367fs | RS555607708 | 48 | Intermediate | Positive | Bilateral DCIS |
| frameshift deletion | CHEK2:NM_007194:exon12:c.1262delT:p.L421fs | NOVEL | 49 | High | Positive |  |
| frameshift insertion | CHEK2:NM_007194:exon12:c.1368dupA:p.S456fs | NOVEL | 41 | High | Negative | Yes: contralateral LCIS |
| frameshift deletion | CHEK2:NM_007194:exon3:c.401_402del:p.D134fs | NOVEL | 42 | High | Positive |  |
