# Supplementary material for: Frequency of pathogenic germline variants in BRCA1, BRCA2, PALB2, CHEK2 and TP53 in ductal carcinoma in situ diagnosed in women under the age of 50 years
Source: Breast Cancer Res. 2019 May 6;21:58. doi: 10.1186/s13058-019-1143-y (PMC6501320; doi:10.1186/s13058-019-1143-y)
Supplement: Supplementary file 8 — PALB2 pathogenic variants in cases. (DOCX 19 kb) [file 13058_2019_1143_MOESM8_ESM.docx]

Additional File 8: *PALB2* pathogenic variants in cases

| **Type of mutation** | **Details** | **ID** | **Age** | **Grade** | **ER status** | **Bilateral** |
| --- | --- | --- | --- | --- | --- | --- |
| stopgain | PALB2:NM_024675:exon13:c.C3549G:p.Y1183X, | RS118203998 | 46 | High | Positive |  |
| frameshift deletion | PALB2:NM_024675:exon4:c.508_509del:p.R170fs, | NOVEL | 44 | High | Positive |  |
| frameshift deletion | PALB2:NM_024675:exon4:c.833delT:p.L278fs, | NOVEL | 41 | High | MISSING |  |
| frameshift insertion | PALB2:NM_024675:exon5:c.2324dupA:p.Q775fs, | NOVEL | 39 | High | Positive |  |
| stopgain | PALB2:NM_024675:exon10:c.G3113A:p.W1038X, | RS180177132 | 43 | Intermediate | Positive |  |
| stopgain | PALB2:NM_024675:exon10:c.G3113A:p.W1038X, | RS180177132 | 49 | High | Positive | Yes |
