# Supplementary material for: Frequency of pathogenic germline variants in BRCA1, BRCA2, PALB2, CHEK2 and TP53 in ductal carcinoma in situ diagnosed in women under the age of 50 years
Source: Breast Cancer Res. 2019 May 6;21:58. doi: 10.1186/s13058-019-1143-y (PMC6501320; doi:10.1186/s13058-019-1143-y)
Supplement: Supplementary file 9 — BRCA1 pathogenic variants in cases. (DOCX 18 kb) [file 13058_2019_1143_MOESM9_ESM.docx]

Additional File 9: *BRCA1* pathogenic variants in cases

| **Type of variant** | **Details** | **ID** | **Age** | **Grade** | **ER status** |
| --- | --- | --- | --- | --- | --- |
| frameshift deletion | BRCA1:NM_007294:exon3:c.114_115del:p.K38fs | NOVEL | 49 | Low | Negative |
| frameshift deletion | BRCA1:NM_007294:exon10:c.3750delG:p.E1250fs | NOVEL | 38 | High | Positive |
| frameshift deletion | BRCA1:NM_007294:exon11:c.4156_4160del:p.S1386fs | NOVEL | 40 | High | Negative |
| stopgain | BRCA1:NM_007294:exon12:c.C4327T:p.R1443X | RS41293455 | 49 | High | Negative |
