# Supplementary material for: Frequency of pathogenic germline variants in BRCA1, BRCA2, PALB2, CHEK2 and TP53 in ductal carcinoma in situ diagnosed in women under the age of 50 years
Source: Breast Cancer Res. 2019 May 6;21:58. doi: 10.1186/s13058-019-1143-y (PMC6501320; doi:10.1186/s13058-019-1143-y)
Supplement: Supplementary file 10 — a: TP53 pathogenic variants in cases. b: TP53 VUS in cases. (DOCX 20 kb) [file 13058_2019_1143_MOESM10_ESM.docx]

Additional File 10a: *TP53* pathogenic variants in cases

| **Type of variant** | **Details** | **ID** | **Age** | **Grade** | **ER status** | **Bilateral** |
| --- | --- | --- | --- | --- | --- | --- |
| stopgain | TP53:NM_000546:exon4:c.G272A:p.W91X | NOVEL | 35 | High | Positive/Negative | Yes |
| nonsynonymous SNV | TP53:NM_000546:exon5:c.G542A:p.R181H | RS397514495 | 45 | Intermediate | Positive |  |
| stopgain | TP53:NM_000546:exon8:c.C916T:p.R306X | RS121913344 | 40 | High | Negative |  |

Additional File 10b: *TP53* VUS in cases

| **Type of variant** | **Details** | **IARC**  **database** | **ID** | **Age** | **Grade** | **ER status** | **FH** | **Present in controls** |
| --- | --- | --- | --- | --- | --- | --- | --- | --- |
| nonsynonymous SNV | TP53:NM_000546  exon8:c.G869A:p.R290H | Yes  VUS | RS55819519 | 47 | High | Positive | 2nd degree BC | Yes - 2 |
| nonsynonymous SNV | TP53:NM_001276695  exon10:c.C915G:p.N305K | No |  | 49 | High | Unknown | 2 x 1st degree BC | No |
| nonsynonymous SNV | TP53:NM_000546  exon8:c.G800A:p.R267Q, | Yes  Damaging | RS587780075 | 44 | High | Unknown | 1st degree BC,  1st degree glioma | No |
