# Supplementary material for: Frequency of pathogenic germline variants in BRCA1, BRCA2, PALB2, CHEK2 and TP53 in ductal carcinoma in situ diagnosed in women under the age of 50 years
Source: Breast Cancer Res. 2019 May 6;21:58. doi: 10.1186/s13058-019-1143-y (PMC6501320; doi:10.1186/s13058-019-1143-y)
Supplement: Supplementary file 11 — Age of onset of DCIS and family history details of women with two pathogenic variants in different genes. (DOCX 19 kb) [file 13058_2019_1143_MOESM11_ESM.docx]

Additional File 11: Age of onset of DCIS and family history details of women with two pathogenic variants in different genes

| **Case** | **Age of onset of DCIS** | **Genes** | **Family History** |
| --- | --- | --- | --- |
| 1 | 38 | BRCA1:  NM_007294:exon10:c.3750delG:p.E1250fs (novel)  BRCA2:  NM_000059:exon11:c.4447delA:p.T1483fs | *Maternal*  Cervical age 37 - 1^st^ DR  Bowel age 59 - 2^nd^ DR |
| 2 | 44 | BRCA2:  NM_000059:exon11:c.4478_4481del:p.E1493fs  CHEK2:  NM_007194:exon11:c.1100delC:p.T367fs | *Paternal*  Bowel age 52 - 1^st^ DR  Breast age 60 - 2^nd^ DR  *Maternal*  Kidney age 50- 2^nd^ DR  Brain age 60- 2^nd^ DR  Breast age 38 - 3^rd^ DR  Breast age 38 - 3^rd^ DR |
| 3 | 30 | BRCA2:  NM_000059:exon11:c.C5682G:p.Y1894X  CHEK2:  NM_007194:exon11:c.1100delC:p.T367fs | *Maternal*  Breast age 46 - 1^st^ DR  Breast age 30 - 2^nd^ DR  Breast age 43 - 2^nd^ DR  Breast age 30 - 3^rd^ DR  Breast age 43 - 3^rd^ DR  Breast age 45 - 3^rd^ DR  Breast age 42 - 3^rd^ DR |
| 4 | 49  (bilateral) | CHEK2:  NM_007194:exon11:c.1100delC:p.T367fs  PALB2:  NM_024675:exon10:c.G3113A:p.W1038X | *Maternal*  Breast age ? - 1^st^ DR  Breast age ? - 2^nd^ DR |
