# Supplementary material for: Frequency of pathogenic germline variants in BRCA1, BRCA2, PALB2, CHEK2 and TP53 in ductal carcinoma in situ diagnosed in women under the age of 50 years
Source: Breast Cancer Res. 2019 May 6;21:58. doi: 10.1186/s13058-019-1143-y (PMC6501320; doi:10.1186/s13058-019-1143-y)
Supplement: Supplementary file 12 — Frequency of variants of unknown significance and DCIS in women < 50 years of age by gene. (DOCX 19 kb) [file 13058_2019_1143_MOESM12_ESM.docx]

Additional File 12: Frequency of Variants of Unknown Significance and DCIS in women <50 years of age by gene

| **Gene** | **Carriers in cases** | **Carriers in controls** | **OR (95% CI)** | **P** |
| --- | --- | --- | --- | --- |
| BRCA2 | 20 | 46 | 1.07 (0.63-1.83) | 0.78 |
| CHEK2 | 17 | 27 | 1.56 (0.85-2.89) | 0.18 |
| PALB2 | 6 | 37 | 0.39 (0.17-0.94) | 0.03 |
| BRCA1 | 11 | 15 | 1.82 (0.83-3.98) | 0.13 |
| TP53 | 3 | 5 | 1.48 (0.35-6.20) | 0.7 |
